# Identifying Frequent Users of an Urban Emergency Medical Service Using Descriptive Statistics and Regression Analyses

**DOI:** 10.5811/westjem.2015.10.28508

**Published:** 2016-01-12

**Authors:** Chenelle Norman, Michael Mello, Bryan Choi

**Affiliations:** * Brown University, Department of Emergency Medicine, Providence, Rhode Island; † Rhode Island Hospital, Injury Prevention Center, Providence, Rhode Island; ‡ Brown University, School of Public Health, Providence, Rhode Island

## Abstract

This retrospective cohort study provides a descriptive analysis of a population that frequently uses an urban emergency medical service (EMS) and identifies factors that contribute to use among all frequent users. For purposes of this study we divided frequent users into the following groups: low- frequent users (4 EMS transports in 2012), medium-frequent users (5 to 6 EMS transports in 2012), high-frequent users (7 to 10 EMS transports in 2012) and super-frequent users (11 or more EMS transports in 2012). Overall, we identified 539 individuals as frequent users.

For all groups of EMS frequent users (i.e. low, medium, high and super) one or more hospital admissions, receiving a referral for follow-up care upon discharge, and having no insurance were found to be statistically significant with frequent EMS use (P<0.05). Within the diagnostic categories, 41.61% of super-frequent users had a diagnosis of “primarily substance abuse/misuse” and among low-frequent users a majority, 53.33%, were identified as having a “reoccurring (medical) diagnosis.” Lastly, relative risk ratios for the highest group of users, super-frequent users, were 3.34 (95% CI [1.90–5.87]) for obtaining at least one referral for follow-up care, 13.67 (95% CI [5.60–33.34]) for having four or more hospital admissions and 5.95 (95% CI [1.80–19.63]) for having a diagnoses of primarily substance abuse/misuse.

Findings from this study demonstrate that among low- and medium-frequent users a majority of patients are using EMS for reoccurring medical conditions. This could potentially be avoided with better care management. In addition, this study adds to the current literature that illustrates a strong correlation between substance abuse/misuse and high/super-frequent EMS use. For the subgroup analysis among individuals 65 years of age and older, we did not find any of the independent variables included in our model to be statistically significant with frequent EMS use.

## INTRODUCTION

There have been several attempts to define the term emergency medical services (EMS) “frequent users” in regards to both frequency and reasons for use. Many studies have defined them as individuals with anywhere between three to nine emergency department (ED) visits in a year.^1^ The Rhode Island State Department of Health defines frequent EMS users as having needed four or more 911 ambulance transports in one calendar year. Squire et. al^2^ and Rucker et. al^3^ found that a greater proportion of individuals frequently transported by ambulance were individuals 65 years or older and Medicare recipients. The most common complaints during transports included chest pain, abdominal pain, and shortness of breath.^4^–^6^

Recently, there has been a growing focus on reducing the number of frequent users as a means of decreasing healthcare costs. The United States Government Accountability Office estimated that a single ground ambulance transport cost from $224 to $2,204 in 2010.^7^ In that same year the Centers for Medicare & Medicaid Services (CMS), the largest single payer for ambulance transports, spent $5.2 billion to cover EMS-related fees and services.^7^ In an attempt to control spending on EMS, especially unreimbursed transport, many cities, states and communities have designed and implemented EMS diversion programs to provide appropriate and cost-effective alternatives for patients with non-emergent and chronic issues. Dunford et al^8^ found that diverting chronic alcoholics with high EMS and ED utilization to non-ED treatment services reduced EMS transports by 50% and resulted in a $5,662 decrease in total monthly EMS charges for those accepting treatment, as well as reducing the “revolving door” effect between jail and frequent ED visits. In Baltimore, Maryland, Rinke et al^9^ showed that improved case management for 10 frequent EMS users decreased each individual’s EMS transports by 32% on average, resulting in an estimated net savings of $14,461.

Providence, Rhode Island, has the state’s highest volume of 911-dispatched EMS calls, the majority of which are handled by the city’s fire department. In 2009, Providence Fire responded to 43,000 calls, most of them medical.^10^ Overall, the department consists of six rescue companies with engine response and transport of a significant number of adult patients to two major hospitals in the city. Both are nonprofit teaching hospitals affiliated with Warren Alpert Medical School of Brown University. The larger of the two hospitals consist of 719 beds and serves as the Level I trauma center for southeastern New England.

### Study Objectives

Although many EMS caregivers can anecdotally identify frequent users of their services, there are few attempts in the literature to systematically examine frequent users’ common features and reasons for use. This knowledge may assist healthcare leaders in developing and implementing appropriate interventions to control EMS overuse and contain healthcare costs in the face of constricting municipal budgets. Our objective was to clearly define and describe characteristics of frequent EMS users in order to provide suggestions for efficient and cost-effective interventions that address the healthcare needs of this vulnerable population.

## METHODS

### Study Sample

We defined frequent users using the Rhode Island State Department of Health’s criteria. The criteria are based on previous literature reporting ED overuse and was reported in the Special Senate Commission to Study Rhode Island Emergency Department Room Diversion in February 2012. Adults with four or more 911-dispatched EMS transports to the ED in 2012 were identified from EMS records (station logs and compiled run reports) provided by Providence Fire. Each identified patient was matched to hospital ED discharge data found within the ED electronic health record (EHR). In order to focus on only individuals with four or more 911 EMS transports, other ED visits resulting from the following transport types were removed: internal (2), car (2), police transported (177), transfer (1), walk-in (2344), unknown (442).

### Extracting Medical Records

With approval from the local institutional review board we electronically extracted the following data from the hospital EHR for all patients identified as frequent users of EMS: date of birth, gender, diagnosis, arrival method, outcome (discharged or admitted), arrival time, departure time, referral for follow-up care, payer name and payer description.

### Cleaning/Preparing Data

We created a de-identified database with patient names replaced with unique identifiers, and dates of birth converted to ages. Ten variables were created for the study: (1) age, (2) gender, (3) total number of ED visits, (4) total number of EMS transports, (5) diagnostic category, (6) insurance status, (7) referral for follow-up care (yes or no), (8) time of EMS transport, (9) number of inpatient admissions, and (10) average length of ED stay in minutes. We created six diagnostic categories for the study:

1. Primarily mental health (MH)
2. Primarily substance abuse (SA)
3. Multiple medical
4. Multiple medical with MH/SA
5. Reoccurring diagnosis
6. Reoccurring diagnosis with SA/MH

For purposes of this study we obtained all discharge data used from ED discharges. Patients categorized under “primarily mental health” or “primarily substance abuse” had discharge diagnosis terms related to MH and SA (Table 1) appear at least 50% of the time for all EMS transports. Cases identified as “multiple medical” had no one diagnostic term show up 50% or more times. And those categorized under “reoccurring diagnosis” had the same or similar diagnostic term show up 50% or more times for all EMS transports. Lastly, patients categorized as “multiple medical with substance abuse/mental health” or “reoccurring diagnosis with substance abuse/mental health” followed the same guidelines as previously mentioned but included more than one SA or MH diagnosis. In situations where the reoccurring diagnosis and a SA or MH diagnosis appear both 50% of the time and an equal number of times, the patient was categorized as “reoccurring diagnosis with substance abuse/mental health.”

Options for insurance status included Medicaid, Medicare, none and other. We could not delineate commercial insurances since it was not always clear from the discharge data provided. Therefore, we combined commercial insurances and other non-Medicare/Medicaid insurances under the category of other. Individuals with a status of “none” had neither an insurance payer nor insurance description listed. For patients with different insurers listed across visits, the patient was classified by insurer most often under payer description.

Referral for follow-up care was coded as “Yes” or “No” and dependent on whether or not an individual was referred to a physician at least once after any EMS-transported ED visit. Time of transport was coded as “AM”, “PM” or “EQUAL.” Individuals with “AM” transports had a majority of transports occur between 7:00AM and 6:59PM. Individuals with “PM” transports had a majority of transports occur between 7:00PM and 6:59AM. And lastly, those categorized as “EQUAL” had an even number of AM and PM transports. We calculated total number of admissions for each patient, and based on the total, patients were assigned to one of the three categories, “0 admissions”, “1 to 3 admissions” or “4 or admissions.” Lastly, average length of stay was calculated for each patient but only for visits that originated from an EMS transport.

### Statistical Analysis

We performed statistical analysis with Stata 12.0 (StataCorp College Station, TX).

Variables were created for gender, age, referral for follow-up care, number of admissions, diagnostic category, and number of EMS transports, insurance status, and average length of visits. The variable age was divided into the following categories: 18–24, 25–34, 35–44, 45–54, 55–64, and 65+. We divided the number of EMS transports into quartiles and defined as “low-frequent users” (4 EMS transports), “medium-frequent users” (5 to 6 EMS transports), “high-frequent users” (7 to 11 EMS transports) and “super-frequent users” (11 EMS transports or more).

Additionally, we conducted a regression analysis with indicator variables, for the outcome/dependent variable (number of EMS transports) and the independent variables. Statistical significance was set at less than alpha 0.05. Additional, analyses included a multinomial logistic regression, which provided relative risk ratios (RRR) and regression analysis for a subset of the population, individuals 65 years of age and older. Lastly, we calculated kappa statistic to test inter-rater agreement for how patients were categorized into diagnostic groups. For the kappa statistic we tested a random 10% sample of patients. Between the two raters there was 96.23% agreement and kappa equaled 0.9491.

## RESULTS

We identified 539 patients and 6,425 individual discharge diagnoses as solely EMS arrivals, out of an initial pool of 643 patients and 9,616 individual discharge diagnoses of frequent users. The number of EMS transports per frequent-user patient ranged from four to 270 in one year. There were more males in all groups of frequent users and the greatest disproportion was found among super-frequent users (68.5% male vs. 31.5% female). Patient ages ranged from 18 to 98 years. In the medium-, high-, and super-frequent user groups the age range most frequently represented was 45–54 years. The most frequent age range in the low=frequent user group was individuals 65 years and older (Table 2).

Thirty percent of all frequent users in this study were most often transported for reasons related to substance abuse and/or misuse, with the majority being alcohol related. Twenty-eight percent fell into the “reoccurring diagnosis” group (same or similar medical condition). Finally, 21% made up the “multiple medical” group.

Among low-frequent users the most common diagnostic category was “reoccurring diagnosis” with no substance abuse/misuse or mental health. A substantial number of low-frequent users received no referral for follow-up care upon discharge (72.38%), and a majority had one to three hospital admissions over the course of a year. Having four or more hospital admissions, a diagnostic category of “primarily substance abuse/misuse,” no insurance status, at least one referral for follow-up care upon discharge, and being 65 years of age and older were all associated with an increased relative risk of EMS transport for these low-frequent users.

The super-frequent user category had a higher frequency of four or more admissions, a higher percentage of individuals with primarily substance abuse/misuse diagnoses (41.61%) and a higher frequency of being referred for follow-up care. Individuals identified as super-frequent users had high RRR pertaining to diagnostic category, hospital admissions and referral for follow-up care. The RRR for those with no hospital admissions relative to those with four or more admissions was 13.67 (95% CI [5.60–33.34]) among super-frequent users compared to medium-frequent users, holding other variables constant. The RRR for those with primarily mental health diagnoses relative to those with primarily substance abuse diagnoses was 5.95 (95% CI [1.80–19.63]) among super-frequent users compared to medium-frequent users holding other variables constant. Finally, the RRR for those with no referral for follow-up care relative to those with at least one referral was 3.34 (95% CI [1.90–5.87]) among super-frequent users compared to medium-frequent users, holding the other variables constant.

Additional analysis looked at use data among individuals 65 years of age and older. No patient characteristics were identified as significant in this unique group.

## DISCUSSION

Frequent EMS users are a diverse group of individuals with a wide array of medical, behavioral and social challenges. We found that the largest group of super-frequent users consisted of individuals with repeated substance misuse diagnoses. This aligns with several studies^3^,^8^,^9^ identifying reasons for frequent use among high ED and EMS users. Despite many challenges, a number of interventions have been implemented to target frequent users with substance abuse issues. One combination of weekly medical and psychological case management resulted in an overall 32% decrease in EMS use among a study of 10 frequent users.^9^ Providing this combination of medical and psychological care may be costly due to the intensity of care and services being provided, but it has the potential to result in better healthcare and perhaps long-term savings in healthcare costs. This merits further study.

The next largest groups of frequent users we studied presented for recurrent medical conditions such as chronic obstructive pulmonary disease (COPD) and chronic abdominal pain. Some of these conditions may be better managed with improved primary care and self-management. The Cochrane Collaboration reviewed a number of studies pertaining to COPD self-management and found that with improved self-management there was a reduction in at least one hospital visit in one year.^11^ Still, because of the lack of randomized controlled trials and the potential for a number of external factors to influence health outcomes Cochrane did not provide a definitive conclusion on how self-management impacts COPD outcomes. In another review investigating the outcomes of self-management and CHF, researchers found that among 857 patients, self-management care reduced “all-cause” hospital readmissions, as well as “heart failure” readmissions, and resulted in a savings of $1,300 to $7,515 per patient per year.^12^

Primary care is another important component to improving medical outcomes and has become the focus of a number of attempts to reduce EMS overuse. Community paramedicine or mobile integrated health (CP/MIH) is one model of community-based healthcare that could potentially reduce costs and transport. The model was developed to use EMTs with additional training to fill in healthcare gaps in collaboration with services such as home care, visiting nurses, or primary care access to address non-emergent conditions in a non-transport capacity.^13^

Referral for follow-up care actually increased among individuals whose intensity of EMS use increased. Although it is difficult to extract the reasons for referrals from the limited data provided, it is possible that more referrals may indicate a sicker population. The same may be true for hospital admissions. Results from our data show that high- and super-frequent users have the highest number of hospital admissions with admissions increasing with increased EMS utilization. In addition, increased hospital admissions significantly increased the risk of being a frequent user.

Men tended to make up a higher percentage of frequent users than women with the percentage growing with increasing EMS use. Men tend to have higher incidence of risky behavior (i.e. substance abuse/misuse and dangerous social/physical activities) compared to women, which may explain why men made up a larger percentage of high- and super-frequent users.^14^

The majority of frequent users were Medicare recipients. This came as a surprise since the majority of frequent users were under 65. However, it is possible that a number of individuals in our study were disabled, which would also allow them to qualify for Medicare.

Lastly, among all levels of EMS utilization, the largest number of frequent users fell between the ages of 45 and 55. The highest percentage of elderly (greater than 65 years of age) was found in the low- and medium-frequent user category. Frequent transports may be a result of the elderly being a sicker population or having a number of comorbidities.

In order to improve care for frequent users interventions may require components of both primary/preventative care and self-management education, involvement of social work and case management resources, and increased use of EMTs trained as community paramedics to provide care in the field. Future research needs to explore unique sub-populations of interest (i.e. elderly, specific reoccurring diagnoses, etc.) for potential tailored strategies to decrease EMS utilization.

## LIMITATIONS

There are a number of limitations to this study. Many of the diagnoses provided in the EHR discharge data were actually symptoms rather than true medical conditions. This made it difficult to identify frequent users’ true underlying medical conditions. In addition, the ED discharge diagnosis may not have been the initial reason for the patient’s call or match up with the chief complaint recorded by the EMS provider. Another limitation was the fact that insurance information was often missing, unclear, or incomplete. Limited insurance data made it difficult to identify whether individuals with no insurance information truly lacked insurance or simply failed to have this information recorded. Testing several independent variables simultaneously increases the probability of obtaining a false-positive correlation and a Type I error.

Lastly, though studies show that age increases the risk of EMS transport, we did not find this effect in our study.^4^ This could be a result of our small subgroup size, 98 patients. We may also have to reconsider the variables used in our model and identify other factors that more strongly influence frequent EMS use among elderly populations, such as having in-home care or lack of personal transportation.

## CONCLUSION

EMS frequent users are a wide spectrum of individuals with an even greater range of underlying reasons for high EMS use. This study demonstrates that among low- and medium-frequent users a majority of patients are using EMS for reoccurring medical conditions. In addition, this study adds to the current literature that illustrates a strong correlation between substance abuse/misuse and high/super-frequent EMS use. Strategies to improve care of chronic medical conditions and direct resource utilization to theses efforts has the potential to reduce the burden of EMS use of this group and possibly improve their health.

## Figures and Tables

**Table 1:** Examples of diagnoses and diagnostic categories in frequent users of emergency medical services.

Diagnostic categories	Diagnoses
Substance misuse/abuse diagnoses	alcohol (intoxication, abuse, withdrawal, dependence, overdose, gastritis), cocaine abuse, heroin, and drug abuse, ingestion, dependence – not defined
Medical diagnoses	abdominal pain, chest pain, headache, pain (i.e. back, neck, arm, hip), abscess, respiratory (asthma, allergy, shortness of breath, infection), COPD, cellulitis, congestive heart failure, cirrhosis, gastrointestinal (colitis, diarrhea, constipation), dental, seizures (epileptic, general, etc.), dizziness, fall, hyper and/or hypoglycemia, syncope, nausea and/or vomiting, weakness, urinary tract infection
Mental health diagnoses	depression, adjustment disorder, disturbance of conduct, stress, anxiety, agitation, bipolar disorder, altered mental status

*COPD*, chronic obstructive pulmonary disease

**Table 2:** Demographic and clinical characteristics for frequent users with varying degrees of EMS utilization in 2012.

Characteristic	Low FrU	Medium FrU	High FrU	Super FrU
Gender				
Male	50.48% (53)	50.99% (77)	54.48% (73)	68.46% (102)
Female	49.52% (52)	49.01% (74)	45.52% (61)	31.54% (47)
Age				
18–24	5.71% (6)	5.30% (8)	2.24% (3)	3.36% (5)
25–34	10.48% (11)	10.60% (16)	8.21% (11)	8.72% (13)
35–44	19.05% (20)	14.57% (22)	17.91% (24)	19.46% (29)
45–54	25.71% (27)	24.50% (37)	33.58% (45)	38.26% (57)
55–64	10.48% (11)	23.84% (36)	20.15% (27)	22.15% (33)
65+	28.57% (30)	21.19% (32)	17.91% (24)	8.05% (12)
Patient category				
Primarily MH	6.67% (7)	9.93% (15)	6.72% (9)	4.03% (6)
Primarily SA	19.05% (20)	23.18% (35)	34.33% (46)	41.61 % (62)
Multiple medical	16.19% (17)	28.48% (43)	24.63% (33)	16.11% (24)
Multiple medical with SA/MH	4.76% (5)	7.95% (12)	9.70% (13)	18.12% (27)
Reoccurring dx	53.33% (56)	29.14% (44)	20.90% (28)	16.11% (24)
Reoccurring dx with SA/MH	0.00% (0)	1.32% (2)	3.73% (5)	4.03% (6)
Insurance status				
Medicare	49.52% (52)	48.34% (73)	41.04% (55)	44.30% (66)
Medicaid	35.24% (37)	42.38% (64)	46.27% (62)	51.01% (76)
Other	9.52% (10)	8.61% (13)	10.45% (14)	4.03% (6)
None	5.71% (6)	0.66% (1)	2.24% (3)	0.67 (1)
Referral for follow-up care (at least one?)				
Yes	27.62% (29)	34.44% (52)	41.79% (56)	51.68% (77)
No	72.38% (76)	65.56% (99)	58.21% (78)	48.32% (72)
Admissions (number of times admitted)				
0	30.48% (32)	25.17% (38)	20.15% (27)	17.45% (26)
1 to 3	56.19% (59)	50.33% (76)	38.81% (52)	36.91% (55)
4+	13.33% (14)	24.50% (37)	41.04% (55)	45.64% (68)

*EMS,* emergency medical services; *dx*, diagnosis; *FrU*, frequent users; *MH*, mental health; *SA*, substance abuse
